# Intracranial Migration of Hardware 16 Years Following Craniosynostosis Repair

**Published:** 2018-01-15

**Authors:** Haripriya S. Ayyala, Ian C. Hoppe, Allison M. Rathmann, Frank S. Ciminello

**Affiliations:** ^a^Division of Plastic Surgery, Department of Surgery, New Jersey Medical School, Rutgers University, Newark; ^b^Advanced Neurosurgery Associates, Rutherford, NJ; ^c^Craniofacial and Pediatric Plastic Surgery, Department of Plastic Surgery, Hackensack University Medical Center, Hackensack, NJ

**Keywords:** craniosynostosis, hardware, surgical complication, transcranial migration

## Abstract

**Introduction:** The techniques used to fixate osteotomized segments of bone have evolved alongside the treatment of craniosynostosis. The use of nonresorbable metal plates and screws offered a method of rigidly stabilizing repositioned segments of bone. Several reports specify the tendency for these fixation systems to “migrate” transcranially. **Methods:** We present a unique case of a patient who initially underwent treatment of multisuture craniosynostosis utilizing titanium miniplates at 6 months of age. At 16 years of age, the patient was returned to the operating room with complaints of pain and contour irregularities, and intracranial migration of the screws and plates was observed. **Results:** The hardware was extracted and the cranium reconstructed. Symptoms resolved and bony contour was improved. **Conclusion:** The craniofacial surgeon considering metal plate fixation in the pediatric population should be aware of the possibility for transcranial plate and screw migration.

Craniosynostosis results from premature fusion of the calvarial sutures and affects 1 in 2500 births worldwide.^1^ Diagnosis is primarily based upon physical examination, although radiographic studies such as 3-dimensional computed tomography (CT) can assist in classification and surgical planning. Treatment includes osteotomies with active reshaping of the cranial vault.

While over the years fixation methods and materials have evolved, the main goal remains to achieve absolute rigid stability to aid in direct bone healing. Titanium microfixation plates and screws are demonstrated to be safe and effective in the adult population but may present complications in the pediatric population. In particular, some surgeons have noted upon reoperation of infants who underwent cranial plate fixation the presence of plate adherence to the inner table of the skull in children and plate and screw isolation on bone islands.^2^^,^^3^ We report a case of transcranial migration of titanium microplates and screws 15 years following surgical correction of craniosynostosis.

## METHODS

**Figure 1:**
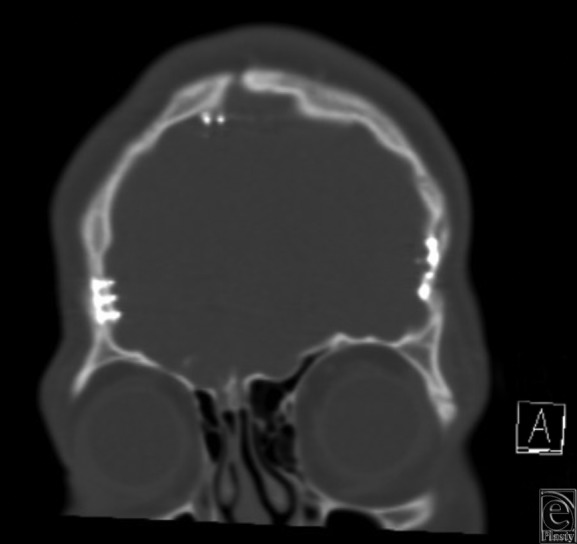
Coronal computed tomographic scan of intracranial extension of hardware.

**Figure 2:**
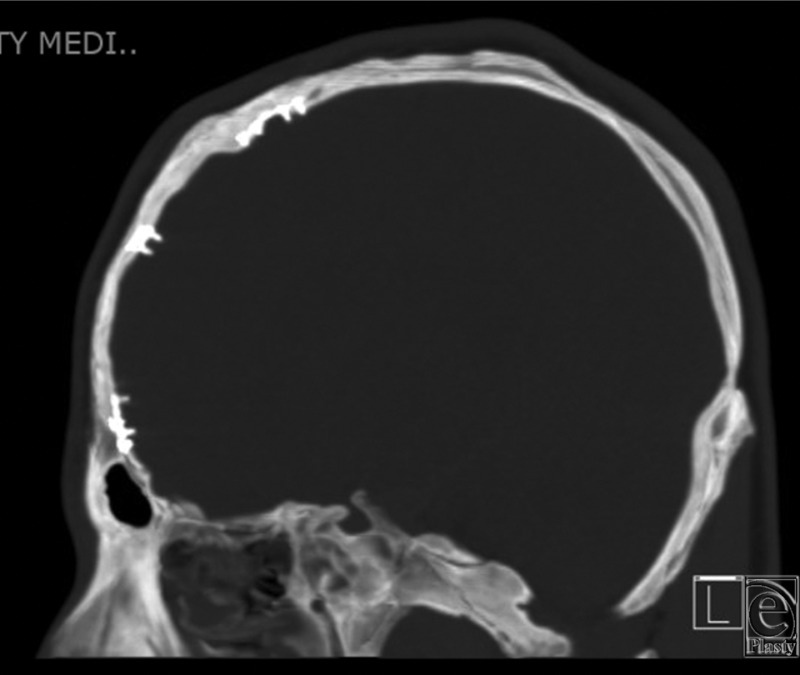
Sagittal computed tomographic scan of intracranial extension of hardware.

**Figure 3:**
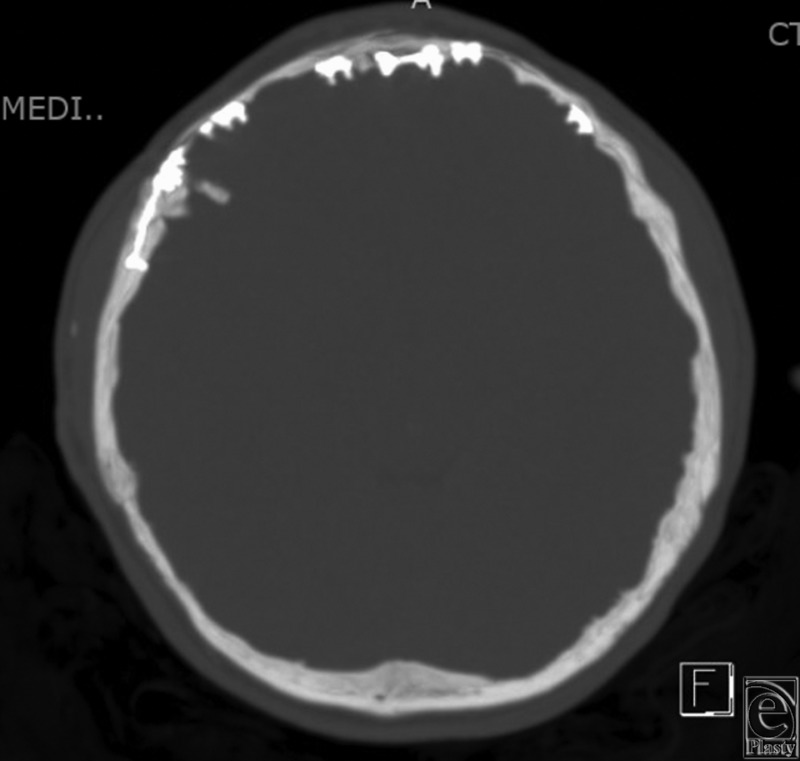
Axial computed tomographic scan of intracranial extension of hardware.

Our patient presented in infancy with right coronal and bilambdoidal craniosynostosis. At 6 months of age, he underwent a fronto-orbital advancement, and at 15 months of age, a posterior vault remodeling procedure. Titanium miniplates and screws were used for stabilization of the bony components. Fifteen years later, the patient presented with symptoms of pain and persistent deformation of the orbit with contour irregularities of the forehead. CT scan revealed intracranial transposition of the screws, penetrating the dura (Figs 1-3).

## RESULTS

**Figure 4:**
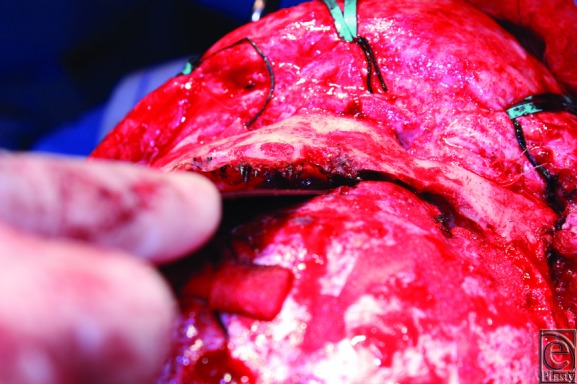
Intraoperative view of the frontal bone with screw extending past the inner table.

**Figure 5:**
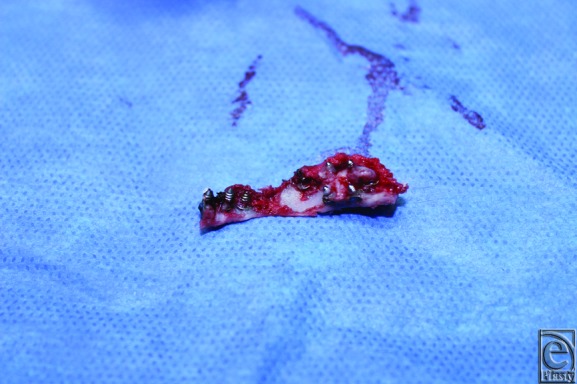
Bone island removed with numerous screws penetrating the inner table.

**Figure 6:**
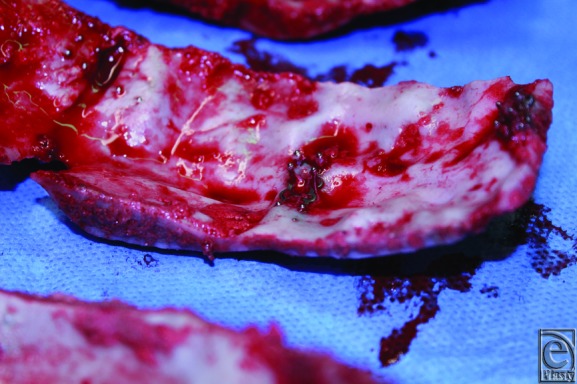
Portion of calvarium demonstrating migration of plates and screws.

The 16-year-old patient was brought to the operating room for hardware removal, possible dural repair, and reconstruction of the forehead and the orbital rim. Intraoperatively, extensive hardware was found extending to the cranionasal junction, up to and beyond the sphenoid wing into the middle cranial fossa. The hardware was imbedded within the bone, with approximately 2 to 3 mm of screw sticking beyond the inner table (Fig 4). The hardware was carefully extracted (Fig 5), and the neurosurgery team repaired a small dural injury. The frontal bone was removed, resected into multiple strips, and contoured (Fig 6). The frontal sinus was obliterated and the bandeau was reconstructed. A series of barrel-stave osteotomies were performed posteriorly. The bony pieces were then rigidly fixated in place. The postoperative course was uneventful, and the patient's symptoms of pain resolved with an improved bony contour.

## DISCUSSION

With the advancement of technology, rigid fixation methods for craniofacial surgery have evolved from stainless steel wiring to metal plates and screws to absorbable plating systems. Since the mid-1990s, there have been several reports describing transcranial migration of fixatives, including wire sutures and metal plates, in children.^2^^-^5 The mechanism by which fixative migration occurs appears to be related to the growth pattern of calvarial bone. The craniofacial skeleton grows by resorption of bone at the inner table and deposition over the outer table. By this process, plates applied to the outer table may “sink” or become trapped as bone is deposited from the inner table to the outer table, eventually resulting in screws reaching the dura.^6^

Alternative fixation methods include utilizing absorbable sutures or bioabsorbable plates. A prospective randomized trial of 16 children with craniosynostosis who underwent bilateral fronto-orbital advancement surgery with either absorbable sutures or rigid fixation (titanium miniplates and absorbable plates) showed that the choice of the fixation material was of minor importance.^7^ Absorbable plating systems manufactured from a combination of polyglycolic and polylactic acids, first introduced in the 1990s, have become increasingly popular. Resorption time varies from 12 to 36 months, depending on the amount of polylactic acid in the system. Studies have documented that the rigidity of absorbable systems is comparable with that of titanium devices.^8^ Long-term sequelae of transcranial migration of plates and screws are not clear. There is little evidence that complications such as cerebrospinal fluid leak, infection, or detectable brain injury occur as a result of plate and/or screw displacement. However, the craniofacial surgeon considering metal plate fixation in the pediatric population should be aware of the possibility for transcranial plate and screw migration.
